# Clinical Condition of the Oral Cavity in the Adult Polish Population below 70 Years of Age after Myocardial Infarction—A Case–Control Study

**DOI:** 10.3390/ijerph19127265

**Published:** 2022-06-14

**Authors:** Marcin Szerszeń, Bartłomiej Górski, Jan Kowalski

**Affiliations:** 1 Department of Prosthodontics, Medical University of Warsaw, 02-097 Warsaw, Poland; 2 Department of Periodontal and Oral Mucosa Diseases, Medical University of Warsaw, 02-097 Warsaw, Poland; bgorski@wum.edu.pl (B.G.); jkowalski@wum.edu.pl (J.K.)

**Keywords:** myocardial infarction, periodontitis, risk factors, tooth loss, treatment needs

## Abstract

According to recent scientific consensus, there is an increasing amount of evidence on the correlation between oral health and cardiovascular disease morbidity. The aim of the present study was to investigate the number of missing teeth, the presence of residual roots with necrotic pulp and teeth with caries, the type of teeth deficiencies, and periodontal status in patients after myocardial infarction (MI). A total of 151 patients after MI and 160 randomly selected controls without history of MI were enrolled in the study. Epidemiological data were collected, and dental examination was performed. Findings showed significantly more women, subjects with lower level of education, lower income, higher percentage of nicotine addiction, more frequent presence of arterial hypertension, diabetes, and obesity than in the study group. Moreover, oral status of the subjects who suffered from MI was inferior to the control group. An average patient from the study group had 11 missing teeth, when compared to four missing teeth in an average control subject (*p* < 0.0001). The majority of patients in the control group had occlusal contacts in intercuspal position in premolars and molars (group A), in contrast to the patients after MI, who had at least one missing supporting zone (group B) (*p* < 0.0001). Severe periodontitis was found in 50.3% of tests and in 30.4% of controls (*p* < 0.0001). A correlation was found between the edentulousness and the risk of myocardial infarction after adjusting for other known risk factors of cardiovascular diseases (OR = 3.8; 95% CI = 3.01–7.21; *p* < 0.0001). This case–control study showed that MI patients had more missing teeth, more residual roots with necrotic pulp, much higher incidence of edentulism and occlusal contacts in intercuspal position in fewer than four occlusal supporting zones, as well as worse periodontal status when compared to healthy subjects without a history of MI. Due to the methodology of unmatched controls, the presented results must be interpreted with caution.

## 1. Introduction

According to the World Health Organization (WHO), oral health is an important indicator of overall health, well-being, and quality of life. There is a proven link between oral diseases and general health [1]. Certain pathological intraoral conditions have an undeniable relationship with systemic health, and some share modifiable risk factors with non-communicable diseases (NCDs) (such as cardiovascular diseases, chronic respiratory diseases, cancer and diabetes), which are among the world’s leading causes of death [2,3,4,5,6,7].

Periodontitis and dental caries are the most common pathological conditions in the oral cavity. Periodontitis is an infectious disease that stems from the interplay between pathogenic agents and host immune reactions. With a global prevalence rate of 40–50%, periodontitis stands as the sixth most common human medical condition [8]. Severe periodontitis affects around 11.2% of the world’s population; its prevalence is associated with age, male gender, low level of education, low socioeconomic status, and tobacco smoking. Dental caries is one of the most pervasive bacterial biofilm-mediated diseases and affects about 2.5 billion people worldwide [9]. Although dental caries is an infectious disease, its pathogenesis is complex and multifactorial and depends among others on dietary and hygienic habits. Regardless of the factors predisposing to the appearance and propagation of carious lesions, the abandonment of treatment causes the loss of mineralized tooth tissues, resulting in infection of the tooth pulp with all subsequent endodontic symptoms, such as, periapical inflammatory lesions. Both periodontitis and caries might lead to tooth loss if left untreated. Tooth deficiency is a form of disability, that may affect dietary intake and nutritional status, leading to overweight or obesity [10]. Moreover, functional impairment in the chewing ability and esthetics, which depend on the oral status, lead to oral health-related quality of life issues for affected patients [11,12].

Cardiovascular diseases (CVD) constitute the leading cause of death worldwide, leading to 17.3 million deaths in 2013 and a predicted 23.6 million deaths in 2030 [13]. In Europe, CVD are responsible for 3.9 million deaths (45%). Numerous studies exist, reporting independent association between severe periodontitis or endodontic pathologies and CVD [4,8]. Considering the relatively high rate of incidence, mortality and disability, myocardial infarction (MI) represents the most important cardiovascular event. Risk factors, such as advanced age, male sex, smoking, obesity, diabetes, low socioeconomic status are considered the common denominators underpinning the relationship between MI and oral health. However, a recent meta-analysis of cohort studies suggested that periodontitis is independently associated with MI (odds ratio 1.13, 95% CI 1.04–1.21), especially in women [14]. In another study, each missing tooth was related to an approximately 1% increase in MI (hazard ratio 1.010; 95% CI 1.007–1.014). Having more than five missing teeth considerably increased the risk of harmful cardiovascular outcomes, and even a small number of missing teeth (one to four) was associated with an increased risk of MI. This association was particularly strong among younger subjects (below 65 years of age) and those with periodontitis [15]. Advanced dental caries has been related to an increased occurrence of coronary heart disease, with a dose–response relationship being observed in middle-aged subjects [16]. Quite recently, untreated caries, periapical lesions, and root fillings, depending on age, were found to be significantly associated with a first MI [17].

The aim of this study was to assess oral health on the basis of tooth loss, presence of residual roots with necrotic pulp, presence of teeth with caries, type of tooth deficiencies, and periodontal status in patients below 70 years of age who have suffered from MI. A further goal was to identify those parameters of oral status that may be potentially associated with the incidence of MI. To our knowledge, this is the first study to investigate the types of tooth deficiencies, and subsequently the characteristics of prosthetic treatment needs, in patients with a history of MI.

## 2. Materials and Methods

The study was carried out in the First Department of Cardiology and in the Department of Periodontal and Oral Mucosa Diseases of the Medical University of Warsaw in accordance with ethical standards from the Declaration of Helsinki. All subjects granted their consent to participate in the project by signing the informed consent form. The study protocol was approved by the Bioethics Committee at the Medical University of Warsaw (approval no. KB-145/2011). STROBE guidelines were followed while conducting and reporting the study [18].

A total of 151 patients (35 females and 116 males) with an average age of 55.1 (±8.0) years were included in the study group; all participants were hospitalized in the First Department of Cardiology of the Medical University of Warsaw due to recent MI on the second or third day of hospitalization. MI was diagnosed in accordance with the criteria of the European Society of Cardiology [19]. The controls were selected by the Ministry of Internal Affairs and Administration from the randomly selected adult population of Poles who reported at the Department of Periodontal and Oral Mucosa Diseases of the Medical University of Warsaw. Adults below 70 years of age were allocated to these two groups, whereas only individuals who did not have a history of MI were qualified to the control group. The exclusion criteria were as follows: (1) neoplastic disease; (2) rheumatic disease; (3) autoimmune disease; (4) chronic liver disease; (5) chronic renal disease stage 4 and 5; and (6) history of stroke.

The following data were collected: (1) income per family member per month (low < 200 EUR, medium 200–360 EUR, high > 360 EUR); (2) education (primary, secondary and higher); (3) smoking (current, in the past, none); (4) arterial hypertension (systolic blood pressure ≥ 140 mm Hg or diastolic blood pressure ≥ 90 mm Hg in three consecutive measurements carried out at 5 min intervals, or use of antihypertensive drugs); (5) diabetes (fasting blood glucose level > 126 mg/dL or use of applicable medication), (6) body mass index (BMI) was determined by dividing the body weight (kg) by height (m^2^), BMI 25–29.9 kg/m^2^ was defined as overweight, and BMI ≥ 30 kg/m^2^ as obesity.

Dental examination was performed by a single calibrated dentist (BG) and included the following factors: (1) the number of teeth present in the oral cavity, third molars were not considered, (2) the number of missing teeth, (3) the number of residual roots with necrotic pulp, (4) the number of teeth with caries detected in clinical examination, (5) the type of teeth deficiencies according to the Eichner classification describing the quality of contacts between teeth in maxilla and mandible (A: intermaxillary contact in four occlusal supporting zones; A1: two full dental arches; A2: one full dental arch, one dental arch with interdentally missing teeth; A3: two dental arches with interdentally missing teeth; B: intermaxillary contact, not in all occlusal supporting zones; B1: intermaxillary contact in three occlusal supporting zones; B2: intermaxillary contact in two occlusal supporting zones; B3: intermaxillary contact in one occlusal supporting zone; B4: intermaxillary contact apart from occlusal supporting zones; C: no intermaxillary contacts; C1: two dental arches with residual dentition; C2: one dental arch with residual dentition, one edentulous arch; C3: two edentulous arches) [20]; (6) periodontal status categorized on the basis of definition by the 2018 EFP/AAP case definition, a participant was a periodontitis case if: interdental clinical attachment level (CAL) ≥ 2 non-adjacent teeth, or buccal or oral CAL ≥ 3 mm with probing pocket depth) PPD > 3 mm is detectable at ≥2 teeth. Then, periodontitis staging was defined according to presence and stage [21]. For the staging, interdental CAL at the site of greatest loss of 1–2, 3–4 and ≥5 mm were considered as mild (stage 1), moderate (stage 2), and severe (stage 3 and stage 4), respectively. To distinguish stage 3 from stage 4, the number of lost teeth due to periodontal reasons was evaluated; if patient lost up to 4 teeth, stage 3 was diagnosed, if patient lost at least 5 teeth due to periodontal reasons then the diagnosis of periodontitis stage 4 was set. Other clinical situations were defined as no periodontitis) [21]. A total of 10 non-study patients with periodontitis were recruited for the calibration exercise. The designated examiner recorded full-mouth PPD and CAL with an interval of 24 h between recordings. Calibration was accepted when ≥90% of the recordings could be reproduced within a difference of 1.0 mm and an exact agreement was repeated in 75% of measurements.

A statistical analysis of the collected data were performed using PQStat v. 1.4.4 (PQStat Software, Poznań, Poland). The χ^2^ test was used for categorical variables, the Mann–Whitney U test was used for continuous variables. *p* ≤ 0.05 was considered statistically significant. Models considering the most important MI risk factors, such as age, sex, diabetes, hypertension, smoking, BMI, education, and income, were constructed. Due to the randomness in the designation of the control group and the similarities and differences resulting from preliminary statistical studies regarding socioeconomic and basic health indicators, the sample size was checked according to the Open-Source Statistics for Public Health Sample Size for Unmatched Case Control Study module [22]. Therefore, multivariate analysis was performed using logistic regression, by calculating the odds ratio (OR) of MI and 95% confidence interval (CI) depending on the number of lost teeth, the number of residual roots with necrotic pulp, the number of teeth with caries, the type of tooth deficiency, and periodontal status. These variables were converted into dichotomous forms, such as over 10 extracted teeth, the presence of residual roots with necrotic pulp, the presence of teeth with caries, tooth deficiency group C according to Eichner, and the diagnosis of severe periodontitis (stage III and stage IV), respectively.

## 3. Results

### 3.1. Sociodemographic and Medical Characteristics of Groups

Socioeconomic and medical characteristics showed significant differences between the study group and the control group. The collected data revealed lower level of education, lower income, higher percentage of nicotine addiction, more frequent presence of arterial hypertension, diabetes, and obesity in the subjects who suffered from MI. The above-mentioned data subjected to Mann–Whitney and χ^2^ tests differed, reaching differences of *p* < 0.5, with the exception of one indicator for diastolic blood pressure. Table 1 shows the sociodemographic and medical characteristics of the analyzed groups.

### 3.2. Oral Characteristics

The oral status of the examined patients is depicted in Table 2. The results clearly indicate greater tooth loss and greater number of residual roots, as well as a significantly worse state of periodontal tissues, in the study group. Almost half of the individuals (49.7%) from the study group were classified as having the most advanced stage of periodontitis. The edentulousness rate was also significantly higher in patients who suffered from MI. In the study group, a higher percentage of patients with quantitative deficiencies was found, when compared to the control group. Statistically significant differences were demonstrated for all comparisons regarding the number of teeth present in the oral cavity. The most striking difference was observed in the percentage of subjects who lost more than 20 teeth—the number of those individuals doubled in the study group.

### 3.3. Type of Teeth Deficiencies

Using the occluso-morphological classification according to Eichner [20], the study and control groups were analyzed, first of all in terms of assignment to general groups (Table 2). A total of 24 patients from the study group were allocated to group A, while in the control group, 68 patients were classified as category A. In group B, the largest number of patients from both the study and control groups were classified, and their percentages were close to 50% (48% and 50.6%, respectively). The difference in the number of classified patients from the study and control groups was the largest in group C, and amounted to 29.1% (36% for the study group and 6.9% for the control group). Subsequently, both groups were evaluated in more detail with respect to their assignment to the appropriate subgroup of tooth deficiency according to Eichner (A1–3, B1–4, C1–3) (Table 3). As a result, 15.3% of participants were placed in subgroup B4 and a percentage of 14.7% in subgroup B2 in the study group, whereas the largest number of patients from the control group (25%) fell into the category of subgroup A3.

### 3.4. Multivariate Analysis

A significant correlation was shown between the number of lost teeth (>10) and the increased MI risk in logistic regression model considering age, sex, diabetes, arterial hypertension, nicotine addiction and BMI (OR = 2.7; 95% CI = 1.2–5.9; *p* = 0.0151) (Table 4). A correlation between group C of tooth deficiency (edentulousness) and MI risk was statistically significant even after adjustment for education and income (OR = 3.6; 95% CI = 0.4–1.9; *p* < 0.0001). The association between the presence of residual roots, the presence of teeth with caries, periodontitis, and MI were not statistically significant after adjustment for smoking, hypertension, diabetes, BMI, education, and income.

## 4. Discussion

Myocardial infarction, as one of the leading causes of premature death worldwide, has a significant impact on both the sociological and economic aspects of all developing and developed countries. A multitude of evidence indicates that patients with compromised oral health suffer from increased cardiovascular risk. Most known risk factors for MI, such as male sex, smoking, high blood pressure, low education, poor socioeconomic status, diabetes, and obesity, were confirmed by the demographic evaluation of the studied population. Oral health was reflected by several variables: periodontitis, presence of residual roots with necrotic pulp, presence of teeth with caries, number of missing teeth and type of tooth deficiencies. The severity of periodontitis, the number of missing teeth, the number of residual roots, and prevalence of edentulousness were considerably higher in subjects after MI, as compared to the controls. It was clearly demonstrated that patients from the study group not only had fewer teeth, but also their distribution and type of dental deficiencies called for greater extent of prosthetic restorations. The majority of patients after MI were missing at least one supporting zone in premolars and molars (group B). In the control group, on the other hand, the characteristics of deficiencies were limited to qualitative and interdental deficits and almost half of this group had all four occlusal supporting zones (group A). The comparison of individual classes of missing teeth determined increased needs of prosthetic treatment in the study group, which would require extensive fixed partial dentures, or even removable dentures, either partial or complete. In the control group, the majority of patients could be treated with small fixed partial dentures or less traumatic implant rehabilitation.

Poor oral health was previously correlated with MI and mortality. Periodontitis verified by radiographic bone loss was associated with an increased risk for first MI in a case–control study (n = 1610) [23]. Holmlund et al. [24] examined whether parameters of periodontal status, such as number of deepened pockets, bleeding on probing, and number of teeth were related to cardiovascular outcomes in a cohort study (n = 8999) with a median follow up time of 15.8 years. The authors reported that only the number of teeth was significantly associated with incidents of MI, which is in line with our observations. In our study, edentulousness, in particular, but no occurrence of periodontitis, was strongly associated with MI risk. The reason behind this may be that tooth loss reflects greater inflammation and compromised oral health more than periodontitis only. By the same token, a recent meta-analysis of 879,084 subjects pinpointed that an increase of 1 missing tooth was correlated with a 1.5% increase in coronary heart disease risk [25]. Periodontal disease usually does not cause symptoms in early stages, which may explain why its indicators, such as probing pocket depth and tooth mobility, are not strongly correlated with oral health-related quality of life.

In another study, multiple dental caries lesions were related to the risk of cardiovascular diseases in hypertensive patients [26]. Quite similarly, Sebring et al. [17] reported that the number of missing teeth, any primary periapical lesion, and the proportion of root-filled teeth were associated with an increased risk of a first MI. Patients who had suffered a first MI had a larger sum of decayed, missing and filled teeth than the healthy controls. The cases and controls did not differ in terms of number of teeth with caries in our study, which contradicted the findings of the abovementioned studies. As only clinical examination was performed, the periapical status could not be evaluated. Moreover, no relationships between the number of teeth with caries, the number of residual roots with necrotic pulp, and MI were found. This discrepancy might be related to study design, sample size, or study population.

It was reported that retention of teeth is associated with better quality of life [27]. The loss of anterior teeth seriously affects face aesthetics. Patients who have lost posterior tooth support, on the other hand, might lose clenching force as a result of increased loading to the remaining anterior dentition and possibly loss of muscle strength. In one study, the maximum clenching force decreased by 258 N (58 lbs), on average, in subjects with moderate loss of posterior tooth support [28]. In our study, an average patient from the study group had 11 missing teeth, in contrast to four missing teeth for an average control patient. Previous investigations have reported that activity of jaw-closing muscles is determined by the number of occlusal contacts—patients with a larger number of teeth that connect in maximal intercuspation showed higher muscular electromyographic (EMG) activity [29,30]. In our study, nearly 40% of subjects from the study group would require extensive prosthodontic rehabilitation, even though demand for treatment might not be well correlated with objectively determined treatment needs. It is commonly known that the financial consequences of edentulousness treatment, especially as it is not refunded in many public sector healthcare systems, is very high. Similarly, in the case of periodontitis, a recent study estimates total and annual average costs of advanced periodontitis treatment, with or without restorative treatment, at EUR 7154 and EUR 437, respectively [31]. However, the prognosis for removable dentures, especially in the case of tooth-tissue-supported and free-saddle designs, may not be predictable or risk-free [32].

Potential connections between cardiovascular diseases with conditions occurring in the oral cavity have been studied for over 20 years [33]. Oral diseases and MI share common risk factors, such as age, male sex, smoking, diabetes mellitus, and obesity, which may serve as confounding factors [34]. Nevertheless, it is generally agreed that bacterial transfer to the bloodstream and outburst of proinflammatory cytokines are among the most important mechanisms linking chronic inflammation in the oral cavity with initiation and development of atherosclerotic plaque [8]. Bacterial invasion of the root canal space in the presence of necrotic pulp is eventually followed by apical periodontitis [35]. Systemic inflammation, in particular, represents the major underlying mechanisms in this relationship. A vast number of factors secreted by oral pathogens (secretome) might influence the host immune system and the pathogenesis of atherosclerosis [36]. It has previously been shown that periodontitis is associated with endothelial dysfunction, whereas its treatment lowers inflammatory mediators and improves endothelial function [37,38]. By the same token, tooth loss is a simple and impartial variable for the accumulated inflammatory load of oral disease, and is independently connected to cardiac events [15,39]. Impaired dentition and consequent inadequate nutritional intake related to tooth loss have also been claimed to be conducive to increasing cardiovascular mortality, but this relationship was reported to be weak by a systematic review [40]. It has been proven that oral hygiene care might reduce the risk of future cardiovascular events in healthy adults [41]. Frequent tooth brushing was associated with a 9% significantly lower risk of cardiovascular events, whereas regular professional cleaning was shown to reduce this risk by 14%.

Taking everything into account, to the best of the authors’ knowledge, this is the first study investigating not only periodontal status, presence of teeth with caries, and number of missing teeth, but also the type of tooth deficiencies, and subsequently the characteristics of prosthetic treatment needs in patients with a history of MI. The diagnosis of periodontitis was set on the basis of the definition by the 2018 EFP/AAP case definition. Moreover, this is the largest case–control study of its kind to be conducted in Central Europe and in Poland. However, when interpreting the presented data, some limitations must also be considered. First, the control group was randomly selected, and consequently differed massively in terms of sociodemographic and medical characteristics (unmatched controls), and this represents the most important disadvantage. None of the subjects in the study group had a healthy oral cavity, occurrence of periodontitis was very high, and almost every examined patient had at least one carious lesion. Another issue is the predominance of men in the study group and women in the control group. This was taken into account in the statistical analysis, where multivariate analysis was adjusted for sex. Moreover, the presence of periodontitis was not determined by objective measures such as evaluation of oral panoramic images, and the presence of carious lesions was assessed only during clinical examination. Nonetheless, the number of missing teeth in this study was adjudicated easily and objectively by dentists. Lastly, the possibility of unadjusted confounders cannot be ruled out. Patients with better oral health may lead healthier lifestyles, including a well-balanced diet, low salt consumption, moderation in alcohol drinking, adherence to medication, and maintenance of optimal body weight. A casual relationship between evaluated variables cannot be determined by a case–control study design without intervention. Therefore, there is a need for further and more thorough evaluation of the connection between oral health and acute cardiac events in future well-designed prospective trials. Possible modulatory effects on the risk of MI when several oral inflammatory conditions exist simultaneously should also be investigated. Furthermore, longitudinal long-term studies of the population would bring valuable data and help confirm or deny the conclusions drawn from the present observation. Larger groups might possibly result in differences in the other outcomes.

## 5. Conclusions

This case–control study showed that MI patients had more missing teeth, more residual roots with necrotic pulp, much higher incidence of edentulism, and occlusal contacts in intercuspal position in fewer than four occlusal supporting zones, as well as worse periodontal status, when compared to healthy subjects without a history of MI. Adjusted models showed increased risk of MI in proportion to number of lost teeth (>10). The strongest correlation was observed between edentulousness and MI risk even after adjustment for sociodemographic factors. Whether these associations stand for a causal or an indirect relationship conciliated by shared risk factors remains uncertain, especially given that the assessed groups were unmatched.

## Figures and Tables

**Table 1:** Sociodemographic characteristics and cardiovascular risk factors in the study groups.

Parameter		Study Group (n = 151)	Control Group (n = 160)	*p*-Value
Sex, n (%)	female	35 (23.2)	97 (60.6)	<0.0001 ^2^
	male	116 (76.8)	63 (39.4)	
Education, n (%)	higher	28 (18.7)	83 (51.9)	<0.0001 ^2^
	secondary	69 (46.0)	66 (41.2)	
	primary	53 (35.3)	11 (6.9)	
Income, n (%)	<€200	46 (30.7)	20 (12.5)	<0.0001 ^2^
	€200–350	57 (38.0)	42 (26.2)	
	>€350	47 (31.3)	98 (61.2)	
Smoking, n (%)	never	28 (18.7)	94 (58.8)	<0.0001 ^2^
	past	26 (17.3)	37 (23.1)	
	current	96 (64.0)	29 (18.1)	
Hypertension, n (%)	no	35 (23.2)	108 (67.5)	<0.0001 ^2^
	yes	116 (76.8)	52 (32.5)	
Systolic blood pressure, mean ± SD		137.2 ± 21.7	124.6 ± 9.6	<0.0001 ^1^
Diastolic blood pressure, mean ± SD		79.9 ± 12.2	82.3 ± 6.6	0.0837
Diabetes, n (%)	no	118 (78.1)	150 (93.8)	<0.0001 ^2^
	yes	33 (21.85)	10 (6.2)	
BMI, mean ± SD		28.9 ± 5.0	26.0 ± 4.0	<0.0001 ^1^
Body weight, n (%)	overweight	62 (42.2)	63 (39.9)	0.0003 ^2^
	obesity	55 (37.4)	33 (20.9)	

^1^ Mann–Whitney U test; ^2^ χ^2^ test.

**Table 2:** Oral status of the study groups.

Parameter		Study Group (n = 151)	Control Group (n = 160)	*p*-Value
Number of present teeth, median (Q1; Q3)		17 (8; 22)	24 (19; 26)	<0.0001 ^1^
Patients with >10 teeth. n (%)		103 (73.5)	147 (93.6)	<0.0001 ^2^
Patients with >20 teeth, n (%)		44 (31.4)	108 (68.7)	<0.0001 ^2^
Number of lost teeth, median (Q1; Q3)		11 (6; 20)	4 (2; 9)	<0.0001 ^1^
Edentulousness, n (%)		18 (12.0)	4 (2.5)	0.0021
Periodontal status, n (%)	No periodontitis	12 (7.9)	49 (32.5)	<0.0001 ^2^
	Stage I	6 (4.0)	11 (7.3)	
	Stage II	40 (26.5)	50 (33.1)	
	Stage III	39 (25.8)	39 (25.8)	
	Stage IV	37 (24.5)	7 (4.6)	
Tooth deficiency group	A	24 (16.0)	68 (42.5)	<0.0001 ^2^
	B	72 (48.0)	81 (50.6)	
	C	54 (36.0)	11 (6.9)	

^1^ Mann–Whitney U test; ^2^ χ^2^ test. Tooth deficiencies: A: intermaxillary contact in four occlusal supporting zones; B: intermaxillary contact in three to one supporting zones; C: no intermaxillary contact.

**Table 3:** The type of tooth deficiency in subgroups (according to Eichner).

Group	Subgroup	Study Group (n = 151)	Control Group (n = 160)	*p* (Test χ^2^)
A	A1, n (%)	3 (2.0)	10 (6.3)	0.062
	A2, n (%)	3 (2.0)	18 (11.3)	0.001
	A3, n (%)	18 (12.0)	40 (25.0)	0.003
B	B1, n (%)	12 (8.0)	30 (18.8)	0.006
	B2, n (%)	22 (14.7)	19 (11.9)	0.468
	B3, n (%)	15 (10.0)	9 (5.6)	0.150
	B4, n (%)	23 (15.3)	23 (14.4)	0.813
C	C1, n (%)	17 (11.3)	1 (0.6)	<0.001
	C2, n (%)	19 (12.7)	6 (3.8)	0.004
	C3, n (%)	18 (12.0)	4 (2.5)	0.001

Eichner classification: A: intermaxillary contact in four occlusal supporting zones; A1: two full dental arches; A2: one full dental arch, one dental arch with interdentally missing teeth; A3: two dental arches with interdentally missing teeth; B: intermaxillary contact, not in all occlusal supporting zones; B1: intermaxillary contact in three occlusal supporting zones; B2: intermaxillary contact in two occlusal supporting zones; B3: intermaxillary contact in one occlusal supporting zone; B4: intermaxillary contact apart from occlusal supporting zones; C: no intermaxillary contacts; C1: two dental arches with residual dentition; C2: one dental arch with residual dentition, one edentulous arch; C3: two edentulous arches.

**Table 4:** Number of lost teeth, presence of residual roots, teeth with caries, advance tooth deficiency and periodontitis and odds ratio in multivariate analysis of adjusted models of subjects studied.

	Unadjusted		Model I ^1^		Model II ^2^		Model III ^3^	
	OR (95% CI)	*p*	OR (95% CI)	*p*	OR (95% CI)	*p*	OR (95% CI)	*p*
Number of lost teeth	4.2 (2.6–7.1)	<0.0001	4.7 (2.4–9.3)	<0.0001	2.7 (1.3–5.0)	0.0133	2.3 (1.1–4.9)	0.0686
Presence of residual roots	1.24 (0.76–3.25)	0.5443	1.91 (1.13–4.11)	0.0231	0.90 (0.58–2.67)	0.7960	1.0 (0.56–2.32)	0.4781
Presence of teeth with caries	0.95 (0.41–1.09)	0.4216	0.9 (0.57–2.12)	0.6910	1.3 (0.82–3.67)	0.8432	0.9 (0.56–1.667)	0.4394
Tooth deficiency group C	3.84 (2.12–4.21)	<0.0001	4.0 (2.76–6.12)	<0.0001	3.1 (1.56–7.45)	0.0048	3.8 (3.01–7.21)	<0.0001
Periodontitis stage III/IV	3.2 (2.0–5.1)	<0.0001	2.0 (1.5–3.7)	0.0098	1.2 (0.6–2.4)	0.6125	1.0 (0.5–1.9)	0.7451
R^2^	0.69		0.73		0.75		0.76	

^1^ Model I adjusted for age and sex; ^2^ Model II adjusted for age, sex, smoking, hypertension, diabetes, BMI; ^3^ Model III adjusted for age, sex, smoking, hypertension, diabetes, BMI, education, and income; OR: odds ratio, CI: confidence interval, R^2^: coefficient of determination.

## Data Availability

The data are not publicly available.
